# Hypertension among Rural Population in Four States: Sudan 2012

**DOI:** 10.5539/gjhs.v6n3p206

**Published:** 2014-03-30

**Authors:** Siham Ahmed Balla, Asma Abdelaal Abdalla, Taha Ahmed Elmukashfi, Haiedr Abu Ahmed

**Affiliations:** 1 Department of Community Medicine, Faculty of Medicine, University of Khartoum, Sudan

**Keywords:** hypertension, rural, diabetes, predictors, target organ damage, Sudan

## Abstract

**Background:**

Hypertension is emerging as an alarming public-health problem causes organ damage.

**Objectives:**

To identify prevalence of hypertension and predictor factors among rural population in four states in Sudan.

**Methods:**

A community based cross-sectional study was conducted in sixteen rural areas in Sudan during April 2012. A total of 3020 adult were interviewed using structured questionnaire and blood pressure was measured before and after the interview. Hypertension was taken as ≥ 140 mmHg and ≥ 90 mmHg for systole and diastole respectively.

**Analysis:**

Descriptive statistic was presented; Sex and mean of systolic and diastolic blood pressure were tested using ANOVA for individuals on antihypertensive medication. Predictor factors to hypertension were tested by logistic regression.

**Results:**

Prevalence of hypertension among rural population was 15.8%. Overall means of systolic and diastolic blood pressure were 128.6±17.7 and 81.5±11.6 respectively while the means among hypertensive individuals was154.74 ±14.4 and 97.98±8.4 respectively Known hypertensive individuals were 20.1%; out of whom 71.7% were hypertensive and 22.4% have Target Organ Damage. Those on anti-hypertensive medications were 76.4% and normotensive were 55.1%. Individuals having both diabetes and hypertension were 3.3% and 80.2% were hypertensive. Log regression model showed age, smoking, diabetes and family hypertension were predictors of hypertension by 3.6%, 34.9%, 49.7% and 56.8% respectively (*P-value* <0.05).

**Conclusion:**

Prevalence of hypertension among rural Sudan was 15.8%. Family history was the strongest predictor of hypertension.

## 1. Introduction

The mortality due to hypertension (HTN) was accounted to 20%–50% of all deaths and the projected number of adults who will have hypertension by 2025 is 1.56 billion (Kearney et al., 2005; Arslantas et al.,2008). It was reported that the highest prevalence of HTN was in Africa and approximately 80% of deaths in low-middle income countries were due to commonest complication of HTN which is cardiovascular disease (WHO, 2011; 2013).

All published studies about hypertension in Sudan targeted small scale studies for different specific population. A study in some referral clinics in Khartoum had shown cardiac, neurological and renal symptoms were the major presenting complaints (Ahmed, 1991). Hypertension was detected in 18.2% of population with different occupations in Khartoum State and 10.2%were known hypertensive (Sherif, Ahmed, & Homeida, 2008). School based study in Khartoum State have shown 4.9% of obese primary school children in age group 6-12 years were hypertensive (Salman, Kirk, & DeBoer, 2011)

Passive screening program in Northern state in Sudan have shown 28.5% of village inhabitants were known hypertensive and 39.6% were having hypertension after screening (Abdelsatir, Al-Sofi, Elamin, & Abu-Aisha, 2013). Local studies were conducted in urban settings rather than rural and showed high prevalence of target organ damages and almost no data was available from rural areas in states (Suliman, 2011).

### 1.1 Objectives

The objectives of this study were to identify the prevalence of hypertension and known hypertensive in rural population of Sudan and to estimate the strength of contributor factors.

### 2. Materials and Methods

This is a descriptive cross sectional, community bases study carried out in rural population in four states of Sudan. The states were River Nile, Northern, Gazera and Gadarif. Sixteen rural areas were selected within states (clusters). Adult rural male and female were the study population.

A two stage cluster sampling was used. The sample size was calculated from the prevalence equation; n= z^2^ P (1-P)/e^2^ multiplied by (de). To compare between states and individuals with and without hypertension, the prevalence was estimated at 50%. The target sample size considers the different ethnics variability in Sudan and to overestimate the sample size, to reduce the error and to increase the significance.

The design effect (de) for the two stages sampling was 2 and the marginal error (e) was 0.05. The standard deviate (z) was selected at 95% Confidence level.

Thereafter; the target sample size was 3073 adult individuals. The non-response rate was 1.7% resulted in a sample of 3020 individuals (510 households). The four states were purposively selected and the sixteen clusters were randomly allocated (four clusters per state). Simple random sampling was used to select the households within each cluster. Two to five adult individuals were interviewed from each household.

Structured questionnaire was used to interview the study population; it contains the variables of population characteristics, smoking status, known hypertension, known diabetes and presence of complications affected central nervous system, renal and cardiovascular. Calibrated mercury sphygmomanometers were used to measure blood pressure for all study population before and after the interview. The minimum interview time was estimated at 7-10 minutes.

Data collectors were the semifinal medical students in the faculty of medicine, university of Khartoum. They had to conduct a training field activity at the community and primary health care level during their fifth medical year. This activity is part of the community medicine curriculum and social accountability dimension. They were trained on the research questionnaire, calibration of sphygmomanometers and the skills of measuring blood pressure. They resided and interact with the communities in the sixteen rural areas (18 students per cluster) for two weeks during April 2012. For the purpose of this study the following definition was considered:

1) Target Organ Damage (TOD): defined as complications in cardiovascular, nervous and renal systems.
2) Known hypertensive: individuals who were diagnosed as hypertensive and on anti-hypertensive medication or not.
3) Hypertension/Hypertensive individuals: used for the measured blood pressure at the time of data collection which according to the cutoff point ≥140 over ≥90 mmHg.

Data was cleaned, entered and managed in SPSS version 20 and a small stata calculator was used for continuous variables. Descriptive statistics was presented in means and standard deviations regarding age, duration of hypertension and diabetes. The means of systolic and diastolic blood pressure were computed and recoded into the desired cutoff point (more than or equal 140 mmHg over more than or equal 90 mmHg).

Detailed description of population with high and normal blood pressure was presented by states as well as prevalence of known hypertensive and diabetic individuals.

Two sample means comparison calculator in small stata was used to test the significance difference between blood pressure of males and females.

Age, sex, education, occupation, education, marital status, family members with hypertension, diabetes and smoking were tested by binary logistic regression.

ANOVA was used for testing sex in relation to systolic and diastolic blood pressure variations and anti-hypertensive medication.

Permission from the states` ministries of health and states` authorities was obtained prior to the departure of the students. Written consent was obtained from each individual in the households. Individuals who were detected with hypertension were given referral sheet to the rural hospital in the area to follow their status. Known hypertensive Individuals with or without complications was provided adequate information to control their hypertensive status.

## 3. Results

The mean age of the total study population was 46.7±13.2 (Min: 18; Max: 95). The populations in the age group 30-60 years constituted 79.1%. Females were 60.7% and males were 39.3%. The first rank occupation was housewives (49.7%) and freelancers occupy the second rank 21.6% (for example, great merchants, sales of different domestic and none domestic goods, shopkeepers, drivers of private vehicles and travel trucks) Employees and farmers constituted 27 % and 72.9% of the population were having varied degrees of literacy (Table 1).

**Table 1:** Descriptive statistics of the study population in rural Sudan

Descriptive statistics		States				
		River Nile	Gadarif	Northern	Gazera	Total
Age/Years	18-30	1.2%	1.6%	1.7%	1.8%	6.2%
	>30-60	21.1%	17.7%	18.3%	22.0%	79.1%
	>60	3.8%	2.1%	3.9%	5.0%	14.8%
	Total	26.0%	21.3%	23.9%	28.8%	100.0%
Sex	Male	10.7%	8.4%	9.5%	10.7%	39.3%
	Female	15.3%	12.9%	14.4%	18.1%	60.7%
	Total	26.0%	21.3%	23.9%	28.8%	100.0%
Marital Status	Not Married	3.0%	1.2%	3.2%	2.1%	9.4%
	Married (Ever and current)	22.9%	20.1%	20.7%	26.8%	90.6%
	Total	26.0%	21.3%	23.9%	28.8%	100.0%
Occupation	Farmer	4.3%	3.8%	3.8%	1.4%	13.3%
	Employee	4.6%	1.7%	3.6%	3.7%	13.7%
	Housewife	11.3%	11.3%	11.6%	15.5%	49.7%
	Freelancers	5.4%	4.3%	4.3%	7.6%	21.6%
	Retired or no work	0.4%	0.2%	0.8%	0.6%	1.8%
	Total	26.0%	21.3%	23.9%	28.8%	100.0%
Education levels	Not educated	7.8%	5.9%	6.6%	6.8%	27.1%
	Primary	9.7%	10.4%	8.0%	11.4%	39.4%
	Secondary	5.4%	3.4%	6.7%	6.8%	22.2%
	University	2.9%	1.4%	2.5%	3.5%	10.3%
	Postgraduate	0.3%	0.1%	0.2%	0.3%	0.8%
	Total	26.0%	21.3%	23.9%	28.8%	100.0%

The overall means of systolic and diastolic blood pressure were 128.6±17.7 and 81.5±11.6 respectively and the mean blood pressure in females was lower than males, 127.9/80.6 versus 129.6/82.9 respectively; *p-value <0.05* (Table 2).

**Table 2:** Distribution of hypertension and diabetes among population in rural Sudan

Hypertension and Diabetes Status		States▲					
		River Nile	Gadarif	Northern	Gazera	Total	*P*-value **
^¥^ ^ᶋ^Measured Blood Pressure	Hypertension ≥140/≥90	15.3%	17.9%	16.4%	14.4%	15.8%	*P*-value > 0.05
^ᾤ^ Known hypertensive population		19.9%	17.3%	19.3%	23.3%	20.1%	*P*-value <0.05
Hypertension among Known hypertensive individuals		75.6%	72.1%	66.2%	72.3%	71.7%	*P*-value >0.05
*TOD among known hypertensive		5.8%	4.1%	5.3%	7.2%	22.4%	*P*-value >0.05
^ᵿ^Diabetic population		12.6%	9.2%	10.119%	10.4%	10.6%	*P-value> 0.05*
Hypertension among diabetic population		50.5%	54.2%	61.6%	51.1%	53.9%	*P-value > 0.05*
Both diabetic and known hypertensive		4.3%	3.0%	2.8%	3.2%	3.3%	*P-value >0.05*
Hypertension among Both diabetic and known hypertensive		85.3%	73.7%	75.0%	82.1%	80.2%	*P*-value >0.05
Individuals on antihypertensive medications		74.8%	78.2%	76.3%	76.7%	76.4%	*P*-value > 0.05
Hypertension among individuals on anti-hypertensive medication		54.3%	50.0%	41.5%	37.4%	44.9%	*P*-value < 0.05

¥ Overall mean systolic BP 128.6±17.7 and mean diastolic BP 81.5±11.6. Mean blood pressure in females and males was 127.9/80.6 versus 129.6/82.9 respectively (*P*-value <0.05). Mean systolic and diastolic blood pressure among hypertensive individuals was 154.74 ±14.4 and 97.98± 8.4 respectively

ᶋ ANOVA test: mean systolic blood pressure among male and females on anti-hypertension medication is 144.93±16.5 versus 146.09±21.1 respectively (*P*- value > 0.05) and mean diastolic blood pressure is 91.60±11.4 versus 88.87±12.2 respectively (*P*-value < 0.05)

ᾤ Mean duration of hypertension is 6.63±6.86

ᵿ Mean duration of diabetes is 7.9±7.89

* (TOD): target organs damage defined as complications regarding central nervous system, renal and cardiovascular

** P value at 95% confidence level using Chi square test

▲ Percent calculated from sample sizes in states.

Hypertension among study population was 15.8% (Mean systolic and diastolic blood pressure was 154.74±14.4 and 97.98±8.4 respectively) with insignificant variation between states. Known hypertensive population (20.1%) showed significant variations between states with the highest prevalence in Gazera state (23.3%). Duration of the hypertension in known hypertensive individuals ranges from 1 to 50 years (Mean: 6.63±6.86). Known hypertensive population who has hypertension and target organ damage (TOD) was 71.7% and 22.4% respectively (Table 2).

Diabetic individuals accounted to 10.6% of rural populations in states and 53.9% have shown hypertension. The duration of diabetes ranges from 1 to 45 years (Mean 7.9±7.89) (Table 2).

Population known to have both hypertension and diabetes were accounting to 3.3% and 80.2% have hypertension. Individuals on antihypertensive drugs accounted to 76.4% and 44.9% have hypertension. Male and females on medication of anti-hypertension have shown mean systolic blood pressure 144.93±16.5 and 146.09±21.1 respectively (P- value > 0.05) and mean diastolic blood pressure 91.60±11.4 and 88.87±12.2 respectively (*P-value < 0.05)* (Table 2).

Regarding factors contributing to hypertension, logistic regression model retained age, smoking, diabetes and history of family members with hypertension and removed from sex, education, occupation, education and marital status (Table 3).

**Table 3:** Predictors of hypertension

Predictor	Factor contribution	Odd Ratio(*p-value <0.05*)	Confidence Interval
Age	3.7%	1.037	1.029 : 1.044
Smoking	34.9 %	1.349	1.011 : 1.798
Diabetes	49.7%	1.497	1.127 : 1.989
Family History of HTN	56.80%	1.568	1.277 : 1.925

## 4. Discussion

Hypertension, defined as a systolic blood pressure equal to or above 140 mm Hg and diastolic blood pressure equal to or above 90 mm Hg (WHO, 2013), it causes 9.4 million deaths due to its complications worldwide.

In this study; mean systolic and mean diastolic blood pressure was 128.6±17.7 and 81.5±11.6 respectively. In our study; the mean blood pressure in females was lower than males (127.9/80.6 versus 129.6/82.9, *P-value <0.05*). Our findings are similar to studies conducted in rural population of Ghana-west Africa and rural India where the mean blood pressure of females was significantly lower than males (Agyemang, 2006; Kusuma & Das, 2008).

Our study showed the mean systolic blood pressure among male and females on anti-hypertension medication was not significantly different while mean diastolic blood pressure was significantly different. However a study in semi-rural area in Turkey showed no difference of hypertension between males and females (Arslantas, Ayranci, Unsal, & Tozun, 2008) while another study showed significant association with sex (Wamala, Karyabakabo, Ndungutse, & Guwatudde, 2009).

Diastolic blood pressure in rural Sudan was exceeding 80 mmHg which may be due to salty and spicy food habits of Sudanese diet. Different ethnic groups in Africa consume more than 6 grams of salt per day while the recommended intake was 5 to 3 grams per day (He & MacGregor, 2004; Charlton et al.,2005). Reducing salt intake to 4.6 gram per day among hypertensive individuals had shown a reduction of the mean diastolic blood pressure by 2.74 mmHg and by 0.99 mmHg in normotensive population if their daily salt was 4.4 gram (He & MacGregor, 2004).

The prevalence of hypertension in our study is much lower than the prevalence in rural India, rural china and rural Uganda (Kusuma & Das, 2008; Maher, Waswa, Baisley, Karabarinde, & Unwin, 2011; Li et al., 2010; Wamala et al.,2009). Compared to semi urban areas in western Turkey, more than half of the population was hypertensive (Arslantas et al.,2008). The differences could be due the methodology of measuring the blood pressure. The time factor between the two measurements in our study was satisfactory to eliminate any personal fear compared to Uganda and china studies where a single measurement and the 5 minutes apart between measurements may be confounded by biological responses.

The mean duration of hypertensive and diabetic status were 6.63±6.86 years and 7.9±7.89 years respectively which are not different from a study carried out in Gazera state at primary health care clinics (Elsharif et al.,2013). The slight difference may be due to the large sample size in our study.

The prevalence of known hypertensive showed significant variations between states population showing the highest rate in Gazera state (23.3%). This state is ranking the second after Khartoum state and the population is similar to urban modern settings.

The target organ damage was 22.4% among hypertensive population. Hypertension causes end stage renal diseases in 14.3% of patients in Gazera State Hospital in Sudan (M. E. Elsharif & E. G. Elsharif, 2011) and contributes to 75% of cardiovascular diseases among diabetic population (Sowers, Epstein, & Frohlich, 2001). The study showed 71.7% of known hypertensive population has hypertension which reflected poor controlling status and they were at risk of developing complications related to hypertension. Compared to rural china, our hypertensive population was relatively controlling their hypertension status (Li et al.,2010).

Population with diabetes in this study showed 53.9% have hypertension which is similar to the findings in a reviewed data in United States (Ong, Cheung, Man, Lau, & Lam, 2007). The biological characteristics and behavioral lifestyle of diabetic individuals may intervene in controlling their normotensive status (Saydah et al.,2004).

Individuals on anti-hypertensive medication accounted to 76.4%, 55.1% were normotensive and 44.9% were hypertensive. This may reflect some adherence to antihypertensive medication compared to a study conducted among Mozambicans with anti-hypertensive medication showed 39.9% were normotensive (Damasceno et al.,2009). Our study is supported by a study in Kassala state in eastern Sudan showed the compliance of antihypertensive drugs among hypertensive patients was 59.6% where 92% of them were normotensive (Elzubier et al.,2000).

It is worth mention that 3.3% of study population of rural Sudan has both diabetes and hypertension and 80.2% was hypertensive reflecting poor control of blood pressure for both diseases.

However, the prevalence of hypertension, known hypertensive and diabetic population in this study is within the reported ranges reported in sub-Saharan Africa (Dalal et al.,2011).

Logistic regression model retained age, smoking, diabetes and family history of HTN as predictors of hypertension. Our study showed 56.8% chance of having hypertension among individual with positive family history of hypertension. This is similar to findings of a study of records in screened cohort which showed the increase in the prevalence of hypertension as the number of family members with history of hypertension increased (Tozawa et al.,2001).

Diabetes mellitus contributed to the prediction of hypertension by 49.7%. Diabetes is a strong factor associated with coronary artery disease and potentiates the risk of vascular and renal complications if co-exist with hypertension (Lago, Singh, & Nesto, 2007). Diabetes and hypertension are closely interrelated; diabetes mellitus could precede the occurrence of hypertension while studies at early of last decade showed hypertensive individuals were more likely to develop diabetes mellitus than normotensive persons (Sowers et al.,2001).

Our study showed smoking as a predictor of hypertension by 34.9% which is similar to a study carried out in rural community of Tamilnadu in India, it showed smoking as statistically significant predictor of hypertension and diabetes (Radhakrishnan & Balamurugan, 2013)

## 5. Conclusion

Prevalence of hypertension among rural Sudan was 15.8% with insignificant variation between the four states. Gazera state showed the highest prevalence of known hypertensive individuals. The mean diastolic blood pressure among known hypertensive on anti-hypertensive medication is significantly higher in males than females raising new information for further researches in Sudan. Family history was strong predictor for hypertension.
